# Existence and uniqueness result for a class of mixed variational problems

**DOI:** 10.1186/s13660-018-1686-y

**Published:** 2018-04-20

**Authors:** Caijing Jiang

**Affiliations:** College of Sciences, Guangxi University for Nationalities, Nanning, P.R. China

**Keywords:** 47J20, 47J22, 49J53, Mixed variational problem, Variational inequalities, Contact problem, Existence, Saddle-point theory

## Abstract

Our aim in this paper is to investigate the existence and uniqueness result for a class of mixed variational problems. They are governed by two variational inequalities. By applying the saddle-point theory, we obtain the existence of solutions to mixed variational problems. Finally, some frictional contact problems are given to illustrate our main results.

## Introduction

The abstract problem in this paper is a class of mixed variational problems governed by two variational inequalities, with a bilinear function and functional which is convex and lower semicontinuous. Considering such kinds of variational problems sets the functional background in the study of elastic contact problems with unilateral constraints and nonmonotone interface laws. For very recent work, see [1–3]. Our results improve the results in [4–7], which consider a bilinear functional.

Let *X* be a Hilbert space, *Y* a reflexive Banach space and Λ be a nonempty, closed and convex subset *Y*. Let $A:X\rightarrow X$, $B:X\times\Lambda\rightarrow\mathbb{R}$, $\phi :X\to\mathbb{R}$ be given maps to be specified later, and $w,f\in X$ be fixed. We consider the mixed variational problems of following forms.

### Problem 1

Find $(u,\lambda)\in X\times\Lambda$ such that

1 $$\begin{aligned}& (Au,v-u)_{X}+B(v-u,\lambda)+\phi(v)-\phi(u)\ge(f,v-u)_{X}, \quad \forall v\in X, \end{aligned}$$

2 $$\begin{aligned}& B(u-w,\mu-\lambda)\le0,\quad\forall\mu\in\Lambda. \end{aligned}$$

Recently, a lot of work was devoted to the modeling in contact mechanics. Weak formulations of contact problems involve the theory of variational inequalities and the theory of saddle-point problems; see e.g. [7–13]. The purpose of this paper is to investigate the weak solvability of a unilateral frictionless contact problem using a technique with dual Lagrange multipliers. The weak formulations with dual Lagrange multipliers allow one to write efficient algorithms in order to approximate the weak solutions. In the present work, the behavior of the materials is described by using the subdifferential of a proper, convex, lower semicontinuous functional and the contact is modeled with Signorini’s condition with zero gap. The results extend and improve the results obtained in [5, 6], where a unilateral frictionless contact model for nonlinearly elastic materials is analyzed.

In this paper, by applying the saddle-point theory, we obtain the existence of solutions to the mixed variational problems. The main results are that Problem 1 has a unique solution $(u,\lambda)\in X\times \Lambda$ and the solution $(u,\lambda)$ is Lipschitz continuous with respect to *f* and *ω*. Then we apply them to find that our model has a unique weak solution and the weak solution is Lipschitz continuous with respect to the data.

The rest of this paper is organized as follows. In Section 2, we will introduce some useful preliminaries and necessary materials. Section 3 is devoted to proving the existence and uniqueness results. In the last section, we give some examples of friction contact problems to illustrate our main results.

## Preliminaries

In this section, we will introduce some basic preliminaries which are used throughout this paper. Denote by $\|\cdot\|_{X}$ and $(\cdot ,\cdot)_{X}$ the norm and inner product of the Hilbert space *X* the inner product of *X*, respectively.

### Definition 2

Let *K* be a nonempty subset of a Banach space *X*. A function $f:K\rightarrow\mathbb{R}$ is said to be

(i) convex on *K* if for every finite subset $\{ x_{1},\ldots,x_{n}\}\subset K$ and $\{\lambda_{1},\ldots,\lambda_{n}\} \subset\mathbb{R}_{+}$ such that $\sum_{i=1}^{n}\lambda_{i}=1$ and $\sum_{i=1}^{n}\lambda_{i}x_{i}\in K$, then
  $$ f\Biggl(\sum_{i=1}^{n}\lambda_{i}x_{i} \Biggr)\leq\sum_{i=1}^{n} \lambda_{i}f(x_{i}); $$
(ii) concave on *K* if −*f* is convex on *K*;
(iii) upper semicontinuous (u.s.c.) at $x_{0}$, if, for any sequence $\{x_{n}\}_{n\geq1}\subset K$ with $x_{n}\rightarrow x_{0}$, we have
  $$ \limsup_{n\rightarrow\infty}f(x_{n})\leq f(x_{0}); $$
(iv) lower semicontinuous (l.s.c.) at $x_{0}$, if, for any sequence $\{x_{n}\}_{n\geq1}\subset K$ with $x_{n}\rightarrow x_{0}$, we have
  $$ \liminf_{n\rightarrow\infty}f(x_{n})\geq f(x_{0}). $$
  *f* is said to be u.s.c. (l.s.c.) on *K* if *f* is u.s.c. (l.s.c.) at *x* for all $x\in K$.

We now recall some elements of convex analysis. For more details, we refer to [10, 14, 15].

### Definition 3

Let *A* and *B* be nonempty sets. A pair $(u,\lambda)\in C\times D$ is said to be a saddle point of a functional $\mathcal{L}:C\times D\rightarrow\mathbb{R}$ if

$$ \mathcal{L}(u,\mu)\leq\mathcal{L}(u,\lambda)\leq\mathcal {L}(v,\lambda),\quad \forall v\in C,\mu\in D. $$

In this paper, we apply the following result to prove our existence result.

### Theorem 4

*Let* *X*, *Y* *be reflexive Banach spaces*, *and let* $A\subset X$, $B\subset Y$ *be nonempty*, *closed*, *convex subsets*. *Assume that a functional* $\mathcal{L}:C\times D\rightarrow\mathbb{R}$ *satisfies the following conditions*:

(i) $v\mapsto\mathcal{L}(v,\mu)$ *is convex and l*.*s*.*c*. *for all* $\mu\in D$,
(ii) $\mu\mapsto\mathcal{L}(v,\mu)$ *is concave and u*.*s*.*c*. *for all* $v\in C$.

*Moreover*, *assume that*

(iii) *C* *is bounded or*
  $$ \lim_{\|v\|_{X}\rightarrow\infty,v\in C}\mathcal{L}(v,\mu _{0})=\infty\quad \textrm{for~some } \mu_{0}\in D, $$
(iv) *D* *is bounded or*
  $$ \lim_{\|\mu\|_{Y}\rightarrow\infty,\mu\in D}\inf_{v\in C}\mathcal {L}(v, \mu_{0})=-\infty. $$

*Then the functional* $\mathcal{L}$ *has at least one saddle point*.

We also recall the following definition.

### Definition 5

Let *X* be a Hilbert space and $\varphi:X\to\mathbb{R}$. *φ* is said to be Gâteaux differentiable at $u\in X$, if there exists an element $\nabla\varphi(u)\in X$ such that

$$ \lim_{t\rightarrow0}\frac{\varphi(u+tv)-\varphi(u)}{t}=\bigl(\nabla \varphi(u),v \bigr)_{X},\quad\forall v\in X. $$

The element $\nabla\varphi(u)$ which satisfies the relation above is unique and is called the gradient of *φ* at *u*. $\varphi:X\to\mathbb{R}$ is said to be Gâteaux differentiable if it is Gâteaux differentiable at every point of *X*. In this case, the operator $\nabla\varphi(u): X \to X$ that maps every element $u\in X$ into the element $\nabla\varphi(u)$ is called the gradient operator of *φ*. The convexity of Gâteaux differentiable functions can be characterized as follows.

### Lemma 6

([16, 17])

*Let* *X* *be a Hilbert space and* $\varphi:X\to\mathbb{R}$ *be Gâteaux differentiable*. *Then* *φ* *is convex if and only if*

$$ \varphi(v)-\varphi(u)\ge\bigl(\nabla\varphi(u),v-u\bigr)_{X},\quad \forall v\in X. $$

## Existence and uniqueness result

We will make the following hypotheses to obtain the existence and uniqueness result for Problem 1:

3 $$\begin{aligned}& \left \{ \textstyle\begin{array}{l} A:X\rightarrow X \mbox{ is such that} \\ \quad\mbox{(a) } \|Au_{1}-Au_{2}\|_{X}\leq L_{A}\|u_{1}-u_{2}\|_{X}\\ \quad\phantom{\mbox{(a) }}\mbox{for all } u_{1},u_{2}\in X \mbox{ with } L_{A}> 0,\\ \quad\mbox{(b) } ( Au_{1}-Au_{2},u_{1}-u_{2})_{X}\ge m_{A}\|u_{1}-u_{2}\|_{X}^{2}\\ \quad\phantom{\mbox{(b) }}\mbox{for all } u_{1},u_{2}\in X \mbox{ with } m_{A}> 0, \end{array}\displaystyle \right . \end{aligned}$$

4 $$\begin{aligned}& \left \{ \textstyle\begin{array}{l} B:X\times\Lambda\rightarrow\mathbb{R} \mbox{ is a bilinear function such that} \\ \quad\mbox{(a) } |B(u,\lambda)|\leq L_{B}\|u\|_{X}\|\lambda\|_{Y}\\ \quad\phantom{\mbox{(a) }}\mbox{for all } u\in X, \lambda\in\Lambda\mbox{ with } L_{B}> 0,\\ \quad\mbox{(b) } \inf_{\lambda\in\Lambda,\lambda\neq0_{Y}}\sup_{u\in X,u\neq0_{X}}\frac{B(u,\lambda)}{\|u\|_{X}\|\lambda\|_{Y}}\ge\alpha_{B}\\ \quad\phantom{\mbox{(b) }}\mbox{with } \alpha_{B}> 0, \end{array}\displaystyle \right . \end{aligned}$$

5 $$\begin{aligned}& \left \{ \textstyle\begin{array}{l} \phi:X\rightarrow\mathbb{R} \mbox{ is a function such that} \\ \quad\mbox{(a) } \phi\mbox{ is l.s.c., convex and G\^{a}teaux differentiable},\\ \quad\mbox{(b) } \|\nabla\phi(u_{1})-\nabla\phi(u_{2})\|_{X}\le L_{\phi}\| u_{1}-u_{2}\|_{X}\\ \quad\phantom{\mbox{(b) }}\mbox{for all } u_{1},u_{2}\in X \mbox{ with } L_{\phi}> 0,\\ \quad\mbox{(c) } (\nabla\phi(u_{1})-\nabla\phi(u_{2}),u_{1}-u_{2})_{X}\ge m_{\phi}\| u_{1}-u_{2}\|_{X}^{2}\\ \quad\phantom{\mbox{(c) }}\mbox{for all } u_{1},u_{2}\in X \mbox{ with } m_{\phi}> 0, \end{array}\displaystyle \right . \end{aligned}$$

6 $$\begin{aligned}& 0_{Y}\in\Lambda. \end{aligned}$$

We will prove the following existence and uniqueness result.

### Theorem 7

*Assume that* (3), (4), (5), (6) *are satisfied*. *Then*, *for any* $\omega,f\in X$, *Problem* 1 *has a unique solution* $(u,\lambda)\in X\times\Lambda$.

Firstly, we give the following equivalence which can be deduced in the proof of Theorem 4 in [6].

### Lemma 8

*Problem* 1 *is equivalent to the following problem*:

*Find* $(u,\lambda)\in X\times\Lambda$ *such that*

$$\begin{gathered} (Au,v)_{X}+B(v,\lambda)+\bigl(\nabla\phi(u).v\bigr)=( f,v)_{X},\quad\forall v\in X, \\ B(u-w,\mu-\lambda)\le0,\quad\forall\mu\in\Lambda.\end{gathered} $$

The proof of Theorem 7 is carried out in several steps. Let $\eta\in X$ be given and consider the following auxiliary problem.

### Problem 9

Given $w,f\in X$, find $(u_{\eta},\lambda_{\eta})\in X\times\Lambda $ such that

7 $$\begin{aligned}& ( u_{\eta},v-u_{\eta})_{X}+\frac{m_{A}}{2L_{A}^{2}}B(v-u_{\eta }, \lambda_{\eta})+\phi(v)-\phi(u_{\eta}) \\& \quad\ge\biggl(\frac{m_{A}}{2L_{A}^{2}}f-\frac{m_{A}}{2L_{A}^{2}}A\eta + \eta,v-u_{\eta}\biggr)_{X},\quad\forall v\in X, \end{aligned}$$

8 $$\begin{aligned}& B(u_{\eta}-w,\mu-\lambda_{\eta})\le0,\quad\forall\mu \in\Lambda. \end{aligned}$$

Define now the operator $T:X\rightarrow X$ by

9 $$ T\eta=u_{\eta}\quad\textrm{for } \eta\in X, $$

where $u_{\eta}\in X$ denotes the unique solution of Problem 9.

### Lemma 10

*The operator* *T* *has a unique fixed point*.

### Proof

From Lemma 23 in the Appendix we know that Problem 9 has a unique solution $(u_{\eta},\lambda _{\eta})\in X\times\Lambda$. Let $\eta_{1},\eta_{2}\in X$ and $(u_{\eta_{1}},\lambda_{\eta _{1}}), (u_{\eta_{2}},\lambda_{\eta_{2}})\in X\times\Lambda$ be the unique solutions of Problem 9 corresponding to $\eta _{1}$, $\eta_{2}$, respectively. From (7) we have

$$\begin{gathered} ( u_{\eta_{1}}-u_{\eta_{2}},u_{\eta_{2}}-u_{\eta_{1}})_{X}+ \frac{m_{A}}{2L_{A}^{2}}B(u_{\eta_{1}}-u_{\eta_{2}},\lambda_{\eta _{2}}- \lambda_{\eta_{1}}) \\ \quad\ge \biggl( \eta_{1}-\eta_{2}+\frac{m_{A}}{2L_{A}^{2}}(A \eta_{2}-A\eta _{1}),u_{\eta_{2}}-u_{\eta_{1}} \biggr)_{X}.\end{gathered} $$

From (8) it follows that

$$ B(u_{\eta_{1}}-u_{\eta_{2}},\lambda_{\eta_{2}}-\lambda_{\eta _{1}}) \leq0. $$

Then

$$ \big\| u_{\eta}^{1}-u_{\eta}^{2} \big\| _{X}\leq \bigg\| \eta_{1}-\eta_{2}+\frac{m_{A}}{2L_{A}^{2}}(A \eta_{2}-A\eta_{1})\bigg\| , $$

and hence

$$ \|u_{\eta_{1}}-u_{\eta_{2}}\|_{X}^{2}\leq \| \eta_{1}-\eta_{2}\|_{X}^{2}- \frac{m_{A}}{L_{A}^{2}}( A\eta _{2}-A\eta_{1},\eta_{1}- \eta_{2})_{X}+\frac{m_{A}^{2}}{4L_{A}^{4}}\| A\eta_{2}-A \eta_{1}\|_{X}^{2}. $$

We use the hypothesis (3)(b) to obtain

$$ \|u_{\eta_{1}}-u_{\eta_{2}}\|_{X}^{2}\leq \biggl(1- \frac{3m_{A}^{2}}{4L_{A}^{4}}\biggr)\|\eta_{1}-\eta_{2} \|_{X}^{2}. $$

It is clear that $m_{A}\leq L_{A}$ and hence $0<1-\frac{3m_{A}^{2}}{4L_{A}^{4}}<1$. Consequently,

$$ \|T\eta_{1}-T\eta_{2}\|_{X}\leq \sqrt {1-\frac{3m_{A}^{2}}{4L_{A}^{4}}}\|\eta_{1}-\eta_{2} \|_{X}, $$

which implies that the operator *T* is a contraction. By applying the Banach contraction principle, we deduce that there exists a unique $\eta^{*}\in X$ such that $\eta^{*}=T\eta^{*}$. This completes the proof of the lemma. □

We now have all the ingredients to provide the proof of the main result in this section.

### Proof of Theorem 7

(i) For the existence, let $\eta^{*}\in X$ be the fixed point of the operator *T*. We write (7) and (8) for $\eta=\eta^{*}$ and observe that $u_{\eta^{*}}=T\eta^{*}=\eta^{*}$. Hence, we conclude that the function $(u_{\eta^{*}},\lambda_{\eta ^{*}})\in X\times\Lambda$ is a solution to Problem 1.

The uniqueness of a solution to Problem 1 is proved directly. Let $(u_{1},\lambda_{1}),(u_{2},\lambda_{2})\in X\times \Lambda$ be two solutions. By Lemma 8 we deduce that

10 $$ ( Au_{1}-Au_{2},v)_{X}+B(v, \lambda_{1}-\lambda_{2})+\bigl( \nabla\phi (u_{1})- \nabla\phi(u_{2}),v\bigr)_{X}= 0,\quad \forall v\in X. $$

Taking $v=u_{1}-u_{2}$ in the above inequality we obtain

11 $$ ( Au_{1}-Au_{2},u_{1}-u_{2})_{X}+B(u_{1}-u_{2}, \lambda_{1}-\lambda_{2})+\bigl( \nabla\phi(u_{1})- \nabla\phi(u_{2}),u_{1}-u_{2}\bigr)_{X}= 0. $$

From (2) it follows that

12 $$ B(u_{1}-u_{2},\lambda_{1}- \lambda_{2})\leq0. $$

Combining (4)(b), (5)(c), (11) and (12), we conclude that $u_{1}=u_{2}$. Moreover, from (10) it follows that

$$ B(v,\lambda_{1}-\lambda_{2})=0, \quad\forall v\in X, $$

which implies that $\lambda_{1}=\lambda_{2}$. This completes the proof of the theorem. □

Next, we consider some stability results.

### Theorem 11

*Assume that* (3), (4), (5), (6) *are satisfied*. *If* $(u_{1},\lambda_{1}),(u_{2},\lambda_{2})\in X\times \Lambda$ *are two solutions of Problem* 1 *corresponding to* $f_{1},f_{2}\in X$ *with* $w=0_{X}$, *then there exists* $C=C(L_{A},m_{A},\alpha_{B},L_{\phi})>0$ *such that*

13 $$ \|u_{1}-u_{2}\|_{X}+\| \lambda_{1}-\lambda_{2}\|_{Y}\leq C\| f_{1}-f_{2}\|_{X}. $$

### Proof

From (1) and (2) we have

$$\begin{gathered} ( Au_{1}-Au_{2},u_{2}-u_{1})_{X}+B(u_{1}-u_{2}, \lambda_{2}-\lambda _{1}) \ge ( f_{1}-f_{2},u_{2}-u_{1})_{X}, \\ B(u_{1}-u_{2},\lambda_{2}-\lambda_{1}) \leq 0.\end{gathered} $$

From (3)(b) it follows that

$$ m_{A}\|u_{1}-u_{2}\|_{X}^{2} \leq\|f_{1}-f_{2}\|_{X}\|u_{1}-u_{2} \|_{X}, $$

and hence

14 $$ \|u_{1}-u_{2}\|_{X}\leq \frac{1}{m_{A}}\|f_{1}-f_{2}\|_{X}. $$

Since

$$ B(v,\lambda_{1}-\lambda_{2})= ( f_{1}-f_{2},v)_{X}-( Au_{1}-Au_{2},v)_{X}-\bigl( \nabla \phi(u_{1})- \nabla\phi (u_{2}),v\bigr)_{X},\quad \forall v\in X. $$

from (5)(b) we have

15 $$ \|\lambda_{1}-\lambda_{2}\|_{Y} \leq\frac{L_{A}+L_{\phi}}{\alpha _{B}}\|u_{1}-u_{2}\|_{X}+ \frac{1}{\alpha_{B}}\|f_{1}-f_{2}\|_{X}. $$

Combining (14) and (15) we get (13). □

### Theorem 12

*Assume that* (3), (4), (5), (6) *are satisfied*. *If* $(u_{1},\lambda_{1}),(u_{2},\lambda_{2})\in X\times \Lambda$ *are two solutions of Problem* 1 *corresponding to* $w_{1}, f_{1}\in X$ *and* $w_{2},f_{2}\in X$ ($w_{i}\neq0_{X}$, $i=1,2$), *then there exists* $C_{1}=C_{1}(L_{A},m_{A},L_{B},\alpha_{B},L_{\phi})>0$ *such that*

16 $$ \|u_{1}-u_{2}\|_{X}+\| \lambda_{1}-\lambda_{2}\|_{Y}\leq C_{1}\bigl( \| w_{1}-w_{2}\|_{X}+\|f_{1}-f_{2} \|_{X}\bigr). $$

### Proof

From (1) and (2) we have

$$\begin{gathered} ( Au_{1}-Au_{2},u_{2}-u_{1})_{X}+B(u_{1}-u_{2}, \lambda_{2}-\lambda _{1})\ge( f_{1}-f_{2},u_{2}-u_{1})_{X}, \\ B(u_{1}-u_{2},\lambda_{2}-\lambda_{1}) \leq B(w_{1}-w_{2},\lambda _{2}- \lambda_{1}).\end{gathered} $$

From (3)(c) it follows that

$$\begin{gathered} m_{A}\|u_{1}-u_{2}\|_{X}^{2} \leq\|f_{1}-f_{2}\|_{X}\|u_{1}-u_{2} \| _{X}+L_{B}\|w_{1}-w_{2} \|_{X}\|\lambda_{1}-\lambda_{2}\|_{Y}, \\ \alpha_{B}\|\lambda_{1}-\lambda_{2} \|_{Y}\leq\|f_{1}-f_{2}\| _{X}+(L_{A}+L_{\phi}) \|u_{1}-u_{2}\|_{X},\end{gathered} $$

and hence

17 $$\begin{aligned}& m_{A}\|u_{1}-u_{2} \|_{X}^{2}\leq\frac{1}{2k_{1}}\|f_{1}-f_{2} \| _{X}^{2}+\frac{k_{1}}{2}\|u_{1}-u_{2} \|_{X}^{2}+\frac {L_{B}^{2}}{2k_{2}}\|w_{1}-w_{2} \|_{X}^{2} +\frac{k_{2}}{2}\|\lambda_{1}- \lambda_{2}\|_{Y}^{2}, \\& \|\lambda_{1}-\lambda_{2}\|_{Y}^{2} \leq\frac{2}{\alpha_{B}^{2}}\bigl(\| f_{1}-f_{2} \|_{X}^{2}+(L_{A}+L_{\phi})^{2} \|u_{1}-u_{2}\|_{X}^{2}\bigr), \end{aligned}$$

where $k_{1}$, $k_{2}$ are strictly positive real constants. Choosing $k_{1}$, $k_{2}$ such that

$$ N:=m_{A}-\frac{k_{1}}{2}-\frac{k_{2}L_{A}^{2}}{\alpha_{B}^{2}}>0, $$

we have

$$ \|u_{1}-u_{2}\|_{X}^{2}\leq \frac{1}{N}\biggl(\frac{1}{2k_{1}}\| f_{1}-f_{2} \|_{X}^{2}+\frac{L_{B}^{2}}{2k_{2}}\|w_{1}-w_{2} \|_{X}^{2}\biggr) +\frac{k_{2}}{N\alpha_{B}^{2}}\|f_{1}-f_{2} \|_{X}^{2}, $$

and combining with (17) we get (16). □

## Contact problems

In this section, we consider some elastic frictional problems to illustrate our main results.

The elastic body occupies an open bounded connected set $\Omega\subset \mathbb{R}^{d}$ ($d=1,2,3$) with boundary $\Gamma=\partial\Omega$, assume to be Lipschitz continuous. Assume that Γ consists of three sets $\overline{\Gamma}_{1}$, $\overline{\Gamma}_{2}$ and $\overline{\Gamma}_{3}$, with mutually disjoint relatively open sets $\Gamma_{1}$, $\Gamma_{2}$ and $\Gamma_{3}$, such that $\operatorname{meas}(\Gamma_{1})>0$.

We use the notation $\boldsymbol {x}= (x_{i})$ for a generic point in $\Omega\cup\Gamma$ and $\boldsymbol {\nu }= (\nu_{i})$ for the outward unit normal at Γ. The indices *i*, *j* run between 1 and *d* and, unless stated otherwise, the summation convention over repeated indices is used. The notation $\mathbb{S}^{d}$ stands for the space of second order symmetric tensors on $\mathbb{R}^{d}$. On the spaces $\mathbb{R}^{d}$ and $\mathbb{S}^{d}$ we use the inner products and the Euclidean norms defined by

$$\begin{gathered} \boldsymbol {u}\cdot \boldsymbol {v}=u_{i}v_{i},\qquad \|\boldsymbol {u}\|=(\boldsymbol {u}\cdot \boldsymbol {u})^{1/2} \quad\textrm {for~all } \boldsymbol {u}=(u_{i}),\boldsymbol {v}=(v_{i}) \in\mathbb{R}^{d}, \\ \boldsymbol {\sigma }\cdot \boldsymbol {\tau }=\sigma_{i}\tau_{i},\qquad \|\boldsymbol {\sigma }\|=(\boldsymbol {\sigma }\cdot \boldsymbol {\sigma })^{1/2}\quad \textrm{for~all } \boldsymbol {\sigma }=(\sigma_{ij}), \boldsymbol {\tau }=(\tau _{ij})\in\mathbb{S}^{d},\end{gathered} $$

respectively. For a vector field, notation $u_{\nu}$ and $\boldsymbol {u}_{\tau }$ represent the normal and tangential components of *u* on Γ given by $u_{\nu}=\boldsymbol {u}\cdot \boldsymbol {\nu }$ and $\boldsymbol {u}_{\tau}=\boldsymbol {u}-u_{\nu} \boldsymbol {\nu }$. Also, $\sigma_{\nu}$ and $\boldsymbol {\sigma }_{\tau}$ represent the normal and tangential components of the stress field ***σ*** on the boundary, i.e. $\sigma_{\nu}=(\boldsymbol {\sigma }\boldsymbol {\nu })\cdot \boldsymbol {\nu }$ and $\boldsymbol {\sigma }_{\tau }=\boldsymbol {\sigma }\boldsymbol {\nu }-\sigma_{\nu} \boldsymbol {\nu }$.

The classical model for the process is as follows.

### Problem 13

Find a displacement field $\boldsymbol {u}\colon\Omega\to\mathbb{R}^{d}$ and a stress field $\boldsymbol {\sigma }\colon\Omega\to\mathbb{S}^{d}$ such that

18 $$\begin{aligned}& \boldsymbol {\sigma }=\mathcal{A}\bigl(\boldsymbol {\varepsilon }(\boldsymbol {u})\bigr) \mbox{ in } \Omega, \end{aligned}$$

19 $$\begin{aligned}& \operatorname{Div} \boldsymbol {\sigma }+ \boldsymbol {f}_{0} =0 \mbox{ in } \Omega, \end{aligned}$$

20 $$\begin{aligned}& \boldsymbol {u}= \textbf{0} \mbox{ on } \Gamma_{1}, \end{aligned}$$

21 $$\begin{aligned}& \boldsymbol {\sigma }\boldsymbol {\nu }=\boldsymbol {f}_{2} \mbox{ on } \Gamma_{2}, \end{aligned}$$

22 $$\begin{aligned}& u_{\nu}\leq0,\qquad \sigma_{\nu}+\eta\leq0,\qquad u_{\nu}(\sigma_{\nu }+\eta)=0, \qquad\eta\in\partial\phi( u_{\nu}) \mbox{ on } \Gamma_{3}, \end{aligned}$$

23 $$\begin{aligned}& \left \{ \textstyle\begin{array}{l} \|\boldsymbol {\sigma }_{\tau}\|\leq\zeta,\\ \|\boldsymbol {\sigma }_{\tau}\|< \zeta\Rightarrow \boldsymbol {u}_{\tau}=0,\\ \|\boldsymbol {\sigma }_{\tau}\|= \zeta\Rightarrow\exists\lambda\geq0\mbox{ s.t. }\boldsymbol {u}_{\tau}=-\boldsymbol {\sigma }_{\tau}, \end{array}\displaystyle \right . \mbox{ on } \Gamma_{3}. \end{aligned}$$

Now, we describe (18)–(23) in the following. Equation (18) represents the elastic constitutive law. Equation (19) is the equation of equilibrium, where $\boldsymbol {f}_{0}$ denotes the density of the body forces, (20) is the displacement homogeneous boundary condition which means that the body is fixed on $\Gamma_{1}$, and (21) is the traction boundary condition with surface tractions of density $\boldsymbol {f}_{2}$ acting on $\Gamma_{2}$. Conditions (22) and (23), given on the contact surface $\Gamma_{3}$, represent the contact and the friction law, respectively. Here *ζ* is the friction bound.

In the rest of the paper we use standard notation for Lebesgue and Sobolev spaces and, in addition, we use the spaces *V* and $\mathcal{H}$ defined by

$$ V=\bigl\{ \boldsymbol {v}\in H^{1}\bigl(\Omega;\mathbb{R}^{d}\bigr)| \boldsymbol {v}=0 \textrm{ on } \Gamma _{1}\bigr\} ,\qquad \mathcal{H}=L^{2}\bigl( \Omega;\mathbb{S}^{d}\bigr). $$

Here and below we still denote by ***v*** the trace of an element $\boldsymbol {v}\in H^{1}(\Omega;\mathbb{R}^{d})$. The space $\mathcal{H}$ will be endowed with the Hilbertian structure given by the inner product

$$ (\boldsymbol {\sigma },\boldsymbol {\tau })_{\mathcal{H}}= \int_{\Omega}\sigma_{ij}(\boldsymbol {x})\tau _{ij}(\boldsymbol {x}) \,dx, $$

and the associated norm $\|\cdot\|_{\mathcal{H}}$. On the space *V* we consider the inner product

$$ (\boldsymbol {u},\boldsymbol {v})_{V}=\bigl(\boldsymbol {\varepsilon }(\boldsymbol {u}),\boldsymbol {\varepsilon }(\boldsymbol {v}) \bigr)_{\mathcal {H}},\quad \boldsymbol {u},\boldsymbol {v}\in V, $$

and the associated norm $\|\cdot\|_{V}$. Recall that, since $\operatorname{meas}(\Gamma_{1}) > 0$, it follows that *V* is a real Hilbert space. Moreover, by the Sobolev trace theorem, we have

$$ \|\boldsymbol {v}_{\tau}\|_{L^{2}(\Gamma_{3};\mathbb{R}^{d})}=\|\gamma \boldsymbol {v}\| _{L^{2}(\Gamma_{3};\mathbb{R}^{d})}\leq\|\gamma \|\|\boldsymbol {v}\|_{V}\quad \textrm{for all } \boldsymbol {v}\in V, $$

where $\|\gamma\|$ is the norm of the trace operator $\gamma :V\rightarrow L^{2}(\Gamma_{3};\mathbb{R}^{d})$.

In the study of Problem 13 we assume that the following conditions hold:

24 $$\begin{aligned}& \left \{ \textstyle\begin{array}{l} \mathcal{A}\colon\Omega\times\mathbb{S}^{d}\rightarrow\mathbb {S}^{d} \mbox{ is such that} \\ \quad\mbox{(a) } \mathcal{A}(\cdot, \boldsymbol {\varepsilon }) \mbox{ is measurable on } \Omega \mbox{ for all } \boldsymbol {\varepsilon }\in\mathbb{S}^{d}, \\ \quad\mbox{(b) } \mbox{ there exists } L_{\mathcal{A}}>0 \mbox{ such that } \\ \quad\phantom{\mbox{(b) }} \|\mathcal{A}(\boldsymbol {x},\boldsymbol {\varepsilon }_{1})- \mathcal{A}(\boldsymbol {x},\boldsymbol {\varepsilon }_{2})\|\leq L_{\mathcal{A}}\| \boldsymbol {\varepsilon }_{1}-\boldsymbol {\varepsilon }_{2}\| \\ \quad\phantom{\mbox{(b) }}\mbox{for all } \boldsymbol {\varepsilon }_{1}, \boldsymbol {\varepsilon }_{2}\in\mathbb {S}^{d}, \mbox{a.e. } \boldsymbol {x}\in\Omega,t \in\mathbb{R}_{+}, \\ \quad\mbox{(c) } \mbox{there exists } m_{\mathcal{A}}>0 \mbox{ such that } \\ \quad \phantom{\mbox{(c) }} (\mathcal{A}(\boldsymbol {x},\boldsymbol {\varepsilon }_{1})- \mathcal{A}(\boldsymbol {x},\boldsymbol {\varepsilon }_{2}))\cdot(\boldsymbol {\varepsilon }_{1}-\boldsymbol {\varepsilon }_{2})\geq m_{\mathcal{A}}\|\boldsymbol {\varepsilon }_{1}-\boldsymbol {\varepsilon }_{2}\|^{2} \\ \quad\phantom{\mbox{(c) }}\mbox{for all } \boldsymbol {\varepsilon }_{1}, \boldsymbol {\varepsilon }_{2}\in\mathbb {S}^{d}, \mbox{a.e. } \boldsymbol {x}\in\Omega, \\ \quad\mbox{(d) } \mathcal{A}(\boldsymbol {x}, {\bf{0}}) = {\bf{0}}, \mbox{a.e. } \boldsymbol {x}\in\Omega, \end{array}\displaystyle \right . \end{aligned}$$

25 $$\begin{aligned}& \left \{ \textstyle\begin{array}{l} \phi:\Omega\times\mathbb{R}\rightarrow\mathbb{R}_{+} \mbox{ is such that} \\ \quad\mbox{(a) } \phi(\cdot,r) \mbox{ is measurable } \mbox{for all } r\in\mathbb {R},\\ \quad\mbox{(b) } \phi(\boldsymbol {x},\cdot) \mbox{is l.s.c., convex and G\^{a}teaux differentiable} \mbox{ for a.e. } \boldsymbol {x}\in\Omega,\\ \quad\mbox{(c) } \|\nabla\phi(\boldsymbol {x},r_{1})-\nabla\phi(\boldsymbol {x},r_{2})\|\le L_{\phi }|r_{1}-r_{2}|\\ \quad\phantom{\mbox{(c) }}\mbox{for all } r_{1},r_{2}\in\mathbb{R}, \mbox{a.e. } \boldsymbol {x}\in\Omega\mbox{ with } M_{\varphi}> 0,\\ \quad\mbox{(d) } (\nabla\phi(\boldsymbol {x},r_{1})-\nabla\phi(\boldsymbol {x},r_{2}),r_{1}-r_{2})\ge m_{\phi }|r_{1}-r_{2}|^{2}\\ \quad\phantom{\mbox{(d) }}\mbox{for all } r_{1},r_{2}\in\mathbb{R}, \mbox{a.e. } \boldsymbol {x}\in\Omega\mbox{ with } m_{\varphi}> 0. \end{array}\displaystyle \right . \end{aligned}$$

Finally, we assume that the densities of body forces and surface tractions have the regularity

$$ \boldsymbol {f}_{0}\in L^{2}\bigl(\Omega;\mathbb{R}^{d} \bigr),\qquad \boldsymbol {f}_{2}\in L^{2}\bigl(\Gamma _{2}; \mathbb{R}^{d}\bigr). $$

We define an operator $A:V\rightarrow V$ by

26 $$ (Au,v)_{V}=\bigl\langle \mathcal{A}\bigl(\boldsymbol {\varepsilon }( \boldsymbol {u})\bigr),\boldsymbol {\varepsilon }(\boldsymbol {v})\bigr\rangle _{\mathcal{H}} $$

for all $\boldsymbol {u},\boldsymbol {v}\in V$. Moreover, we define an element $\boldsymbol {f}\in V^{*}$ by

27 $$ \langle \boldsymbol {f},\boldsymbol {v}\rangle_{V}= \langle \boldsymbol {f}_{0}, \boldsymbol {v}\rangle_{L^{2}(\Omega;\mathbb{R}^{d})} +\langle \boldsymbol {f}_{2},\boldsymbol {v}\rangle_{L^{2}(\Gamma_{2};\mathbb{R}^{d})} $$

for all $\boldsymbol {v}\in V$.

Next, we introduce the space of admissible displacement fields *U* defined by

$$ U=\{ \boldsymbol {v}\in V \mid v_{\nu}\le0 \mbox{ a.e. on } \Gamma_{3} \}. $$

Then $(U,(\cdot,\cdot)_{U})$ is a Hilbert space, where

$$ (\boldsymbol {u},\boldsymbol {v})_{U}=(\boldsymbol {u},\boldsymbol {v})_{V},\quad\forall \boldsymbol {u},\boldsymbol {v}\in U. $$

From Proposition 2.1 and Remark 2.2 in [5] we know that $\gamma (U)$ can be organized as a Hilbert space. Let $D^{T}$ be the dual space of $\gamma(U)$. We define $\boldsymbol {\lambda }\in D^{T}$ by

$$ \langle \boldsymbol {\lambda },\gamma \boldsymbol {v}\rangle_{T}=- \int_{\Gamma_{3}}\boldsymbol {\sigma }_{\tau}\cdot \boldsymbol {v}_{\tau}\,dx, \quad\forall \boldsymbol {v}\in U, $$

where $\langle\cdot,\cdot\rangle_{T}$ denotes the duality pairing between $D^{T}$ and $\gamma(U)$. Furthermore, we define $B:U\times D^{T}\rightarrow\mathbb{R}$ by

28 $$ B(\boldsymbol {v},\boldsymbol {\mu })=\langle \boldsymbol {\mu },\gamma \boldsymbol {v}\rangle_{T},\quad \forall \boldsymbol {v}\in U,\boldsymbol {\mu }\in D^{T}. $$

Let us introduce the following subset of $D^{T}$:

29 $$ \Lambda=\biggl\{ \mu\in D^{T}\Big|\langle \boldsymbol {\mu },\gamma \boldsymbol {v}\rangle_{T}\leq \int _{\Gamma_{3}}\zeta|\gamma \boldsymbol {v}|\,dx, \forall \boldsymbol {v}\in U\biggr\} . $$

Obviously, $\boldsymbol {\lambda }\in\Lambda$. Moreover, from (23) it follows that

$$ B(\boldsymbol {u},\boldsymbol {\lambda })= \int_{\Gamma_{3}}\zeta|\gamma \boldsymbol {u}|\,dx $$

and

$$ B(\boldsymbol {u},\boldsymbol {\mu })\leq \int_{\Gamma_{3}}\zeta|\gamma \boldsymbol {u}|\,dx,\quad \boldsymbol {\mu }\in\Lambda. $$

Then, combining (18)–(23), from Problem 4.1 in [5], Problem 13 can be written as the following variational formulation:

### Problem 14

Find $(\boldsymbol {u},\boldsymbol {\lambda })\in U\times\Lambda$ such that

$$\begin{gathered} (A\boldsymbol {u}, \boldsymbol {v}-\boldsymbol {u})_{U}+B(\boldsymbol {v}-\boldsymbol {u},\boldsymbol {\lambda })+\phi(\boldsymbol {v})-\phi(\boldsymbol {u}) = (\boldsymbol {f}, \boldsymbol {v}-\boldsymbol {u})_{U},\quad\forall \boldsymbol {v}\in U, \\ B(\boldsymbol {u},\boldsymbol {\mu }-\boldsymbol {\lambda })\leq0,\quad\forall \boldsymbol {\mu }\in\Lambda.\end{gathered} $$

Now, we give the following existence, uniqueness and dependence results.

### Theorem 15

*Assume that* (24) *and* (25) *are satisfied*. *Then Problem* 14 *has a unique solution* $(\boldsymbol {u},\boldsymbol {\lambda }) \in U\times\Lambda$. *Moreover*, *if* $(\boldsymbol {u}_{1},\boldsymbol {\lambda }_{1}),(\boldsymbol {u}_{2},\boldsymbol {\lambda }_{2})\in U\times\Lambda$ *are two solutions of Problem* 14 *corresponding to* $\boldsymbol {f}^{1},\boldsymbol {f}^{2}\in U$, *then there exists* $C_{2}=C_{2}(L_{\mathcal{A}},m_{\mathcal{A}},L_{\phi},\gamma)>0$ *such that*

30 $$ \|\boldsymbol {u}_{1}-\boldsymbol {u}_{2}\|_{U}+\| \boldsymbol {\lambda }_{1}-\boldsymbol {\lambda }_{2}\|_{D^{T}}\leq C_{2}\big\| \boldsymbol {f}^{1}-\boldsymbol {f}^{2}\big\| _{U}. $$

### Proof

The proof is analogous to Theorem 6.1 in [5]. From (24) and (25), it is easy to verify that (3) and (5) hold. Then, by applying Theorem 7 and Theorem 11, we can obtain the results. □

### Problem 16

Find a displacement field $\boldsymbol {u}\colon\Omega\to\mathbb{R}^{d}$ and a stress field $\boldsymbol {\sigma }\colon\Omega\to\mathbb{S}^{d}$ such that

31 $$\begin{aligned}[b] &\boldsymbol {\sigma }=\mathcal{A}\bigl(\boldsymbol {\varepsilon }(\boldsymbol {u})\bigr) \mbox{ in } \Omega, \\ &\operatorname{Div} \boldsymbol {\sigma }+ \boldsymbol {f}_{0} =0 \mbox{ in } \Omega, \\ &\boldsymbol {u}= \textbf{0} \mbox{ on } \Gamma_{1}, \\ &\boldsymbol {\sigma }\boldsymbol {\nu }=\boldsymbol {f}_{2} \mbox{ on } \Gamma_{2}, \\ &\boldsymbol {\sigma }_{\tau}=0,\qquad u_{\nu}\le0,\qquad\sigma_{\nu}+ \eta\leq0,\qquad u_{\nu }(\sigma_{\nu}+\eta)=0, \qquad\eta\in\partial\phi( u_{\nu}) \mbox{ on } \Gamma_{3}.\end{aligned} $$

Let $D^{S}$ be the dual space of $\gamma(V)$. We define $\boldsymbol {\lambda }\in D^{S}$ by

$$ \langle \boldsymbol {\lambda },\gamma \boldsymbol {v}\rangle_{S}=- \int_{\Gamma_{3}}\sigma _{\nu}v _{\nu}\,dx,\quad \forall \boldsymbol {v}\in V, $$

where $\langle\cdot,\cdot\rangle_{S}$ denotes the duality pairing between $D^{S}$ and $\gamma(V)$. Furthermore, we define $B:V\times D^{S}\rightarrow\mathbb{R}$ by

32 $$ B(\boldsymbol {v},\boldsymbol {\mu })=\langle \boldsymbol {\mu },\gamma \boldsymbol {v}\rangle_{S},\quad \forall \boldsymbol {v}\in U,\boldsymbol {\mu }\in D^{S}. $$

Let us introduce the following subset of $D^{T}$:

33 $$ \Lambda=\bigl\{ \mu\in D^{T}|\langle \boldsymbol {\mu },\gamma \boldsymbol {v}\rangle_{S}\leq 0,\forall \boldsymbol {v}\in U\bigr\} . $$

Obviously, $\boldsymbol {\lambda }\in\Lambda$. Moreover, from (31) it follows that

$$ B(\boldsymbol {u},\boldsymbol {\lambda })=0 $$

and

$$ B(\boldsymbol {u},\boldsymbol {\mu })\leq0,\quad\forall \boldsymbol {\mu }\in\Lambda. $$

Then Problem 16 can be written as the following variational formulation.

### Problem 17

Find $(\boldsymbol {u},\boldsymbol {\lambda })\in V\times\Lambda$ such that

$$\begin{gathered} (A\boldsymbol {u}, \boldsymbol {v}-\boldsymbol {u})_{V}+B(\boldsymbol {\lambda },\boldsymbol {v}-\boldsymbol {u})+\phi(\boldsymbol {v})-\phi(\boldsymbol {u}) = (\boldsymbol {f}, \boldsymbol {v}-\boldsymbol {u})_{V},\quad\forall \boldsymbol {v}\in V, \\ B(\boldsymbol {u},\boldsymbol {\mu }-\boldsymbol {\lambda })\leq0,\quad\forall \boldsymbol {\mu }\in\Lambda.\end{gathered} $$

We have the following result which is analogous to Theorem 6.2 in [5] and Theorem 15.

### Theorem 18

*Assume that* (24) *is satisfied*. *Then Problem* 17 *has a unique solution* $(\boldsymbol {u},\boldsymbol {\lambda }) \in V\times\Lambda$. *Moreover*, *if* $(\boldsymbol {u}_{1},\boldsymbol {\lambda }_{1}),(\boldsymbol {u}_{2},\boldsymbol {\lambda }_{2})\in V\times\Lambda$ *are two solutions of Problem* 17 *corresponding to* $\boldsymbol {f}^{1},\boldsymbol {f}^{2}\in V$, *then there exists* $C_{3}=C_{3}(L_{\mathcal{A}},m_{\mathcal{A}},L_{\phi},\gamma)>0$ *such that*

34 $$ \|\boldsymbol {u}_{1}-\boldsymbol {u}_{2}\|_{V}+\| \boldsymbol {\lambda }_{1}-\boldsymbol {\lambda }_{2}\|_{D^{S}}\leq C_{3}\big\| \boldsymbol {f}^{1}-\boldsymbol {f}^{2}\big\| _{V}. $$

### Problem 19

Find a displacement field $\boldsymbol {u}\colon\Omega\to\mathbb{R}^{d}$ and a stress field $\boldsymbol {\sigma }\colon\Omega\to\mathbb{S}^{d}$ such that

35 $$\begin{aligned}[b] &\boldsymbol {\sigma }=\mathcal{A}\bigl(\boldsymbol {\varepsilon }(\boldsymbol {u})\bigr) \mbox{ in } \Omega, \\ &\operatorname{Div} \boldsymbol {\sigma }+ \boldsymbol {f}_{0} =0 \mbox{ in } \Omega, \\ &\boldsymbol {u}= \textbf{0} \mbox{ on } \Gamma_{1}, \\ &\boldsymbol {\sigma }\boldsymbol {\nu }=\boldsymbol {f}_{2} \mbox{ on } \Gamma_{2}, \\ &\begin{gathered} \boldsymbol {\sigma }_{\tau}=0,\qquad u_{\nu}\le g,\qquad\sigma_{\nu}+ \eta\leq0,\\ (u_{\nu }-g) (\sigma_{\nu}+\eta)=0, \qquad\eta\in\partial \phi( u_{\nu}) \mbox{ on } \Gamma_{3}.\end{gathered}\end{aligned} $$

Here $g:\Gamma_{3}\to \mathbb{R}_{+}$ denotes the gap between the deformable body and the foundation, measured along the outward normal *τ*.

We keep (32) and (33). We assume that there exists $g_{\mathrm{ext}}:\Omega\to \mathbb{R}$ such that

$$\begin{gathered} g_{\mathrm{ext}}\in H^{1}(\Omega),\qquad \gamma' g_{\mathrm{ext}}=0 \mbox{ a.e. on } \Gamma_{1},\\ \gamma' g_{\mathrm{ext}}\ge0 \mbox{ a.e. on } \Gamma \setminus \Gamma_{1},\qquad g=\gamma' g_{\mathrm{ext}} \mbox{ a.e. on } \Gamma\setminus\Gamma_{3},\end{gathered} $$

where $\gamma':H^{1}(\Omega)\to L^{2}(\Gamma)$ is the well-known Sobolev trace operator. Moreover, we assume that the unit outward normal to $\Gamma_{3}$, $\boldsymbol {\nu }_{3}$, is a constant vector.

Then from (35) it follows that

$$ B(\boldsymbol {u},\boldsymbol {\lambda })=B(g_{\mathrm{ext}}\boldsymbol {\nu }_{3} ,\boldsymbol {\lambda }), $$

and by (33) we have

$$ B(\boldsymbol {u},\boldsymbol {\mu })\leq B(g_{\mathrm{ext}}\boldsymbol {\nu }_{3} ,\boldsymbol {\mu }),\quad\forall \boldsymbol {\mu }\in \Lambda. $$

Then Problem 19 can be written as the following variational formulation:

### Problem 20

Find $(\boldsymbol {u},\boldsymbol {\lambda })\in V\times\Lambda$ such that

$$\begin{gathered} (A\boldsymbol {u}, \boldsymbol {v}-\boldsymbol {u})_{V}+B(\boldsymbol {\lambda },\boldsymbol {v}-\boldsymbol {u})+\phi(v)-\phi(u) = (\boldsymbol {f},\boldsymbol {v}- \boldsymbol {u})_{V},\quad\forall \boldsymbol {v}\in V, \\ B(\boldsymbol {u}-g_{\mathrm{ext}}\boldsymbol {\nu }_{3},\boldsymbol {\mu }-\boldsymbol {\lambda }),\quad\forall \boldsymbol {\mu }\in \Lambda.\end{gathered} $$

We have the following result which is analogous to Theorem 6.3 in [5] and Theorem 15.

### Theorem 21

*Assume that* (24) *is satisfied*. *Then Problem* 20 *has a unique solution* $(\boldsymbol {u},\boldsymbol {\lambda }) \in V\times\Lambda$. *Moreover*, *if* $(\boldsymbol {u}_{1},\boldsymbol {\lambda }_{1}),(\boldsymbol {u}_{2},\boldsymbol {\lambda }_{2})\in V\times\Lambda$ *are two solutions of Problem* 20 *corresponding to* $\boldsymbol {f}^{1},\boldsymbol {f}^{2},g_{\mathrm{ext}}\boldsymbol {\nu }_{3},g^{*}_{\mathrm{ext}}\boldsymbol {\nu }_{3}\in V$, *then there exists* $C_{4}=C_{4}(L_{\mathcal {A}},m_{\mathcal{A}},L_{\phi},\gamma)>0$ *such that*

36 $$ \|\boldsymbol {u}_{1}-\boldsymbol {u}_{2}\|_{V}+\| \boldsymbol {\lambda }_{1}-\boldsymbol {\lambda }_{2}\|_{D^{S}}\leq C_{4}\bigl(\big\| \boldsymbol {f}^{1}-\boldsymbol {f}^{2}\big\| _{V}+ \big\| g_{\mathrm{ext}}\boldsymbol {\nu }_{3}-g^{*}_{\mathrm{ext}}\boldsymbol {\nu }_{3} \big\| _{V}\bigr). $$

## Conclusion

In this paper, we study a class of mixed variational problems governed by two variational inequalities with dual Lagrange multipliers. We prove the existence and uniqueness of solution to the mixed variational problem and apply it to obtain the solutions to some frictional contact problems whose weak formulation can be transferred to the mixed variational problem. We point out that considering such kinds of mixed variational problems sets the functional background in the study of many frictional contact problems. To conclude, our results improve many results with bilinear cases and can be further studied.

## Appendix

We will show the solvability of Problem 9 by using saddle-point theory with the functional $\mathcal{L}_{\eta}:X\times\Lambda \rightarrow\mathbb{R}$,

37 $$ \mathcal{L}_{\eta}(u,\lambda)=\frac{1}{2}( u,u)_{X}-\biggl( \frac {m_{A}}{2L_{A}^{2}}f-\frac{m_{A}}{2L_{A}^{2}}A\eta+\eta,u \biggr)_{X}+\frac {m_{A}}{2L_{A}^{2}}B(u-w,\lambda)+\phi(u), $$

where

$$ f_{\eta}=\frac{m_{A}}{2L_{A}^{2}}f-\frac{m_{A}}{2L_{A}^{2}}A\eta+\eta. $$

### Lemma 22

*Problem* 9 *has a solution* $(u_{\eta},\lambda_{\eta})\in X\times\Lambda$ *if and only if* $(u_{\eta},\lambda_{\eta})$ *is a saddle point of the functional* $\mathcal{L}_{\eta}$.

### Proof

For the necessity, let $(u_{\eta},\lambda_{\eta})\in X\times\Lambda $ be a solution of Problem 9. Inequality (8) implies that

$$ \mathcal{L}_{\eta}(u_{\eta},\mu)\leq\mathcal{L}_{\eta}(u_{\eta }, \lambda_{\eta}),\quad\forall\mu\in\Lambda. $$

Moreover, by combining (7) and (37), we have

$$\begin{gathered} \mathcal{L}_{\eta}(u_{\eta},\lambda_{\eta})- \mathcal{L}_{\eta }(v,\lambda_{\eta}) \\ \quad = \frac{1}{2}( u_{\eta},u_{\eta})_{X}- \frac{1}{2}( v,v)_{X}-(f_{\eta},u_{\eta}-v)_{X} +\frac{m_{A}}{2L_{A}^{2}}B(u_{\eta}-v,\lambda_{\eta})+ \phi(u_{\eta })-\phi(v) \\ \quad\le -\frac{1}{2}\|u_{\eta}-v\|_{X}^{2} \leq0.\end{gathered} $$

Therefore,

$$ \mathcal{L}_{\eta}(u_{\eta},\lambda_{\eta})\leq \mathcal{L}_{\eta }(v,\lambda_{\eta}),\quad\forall v\in X. $$

For the sufficiency, let $(u_{\eta},\lambda_{\eta})\in X\times \Lambda$ be a saddle point of functional $\mathcal{L}_{\eta}$. Note that

$$ \mathcal{L}_{\eta}(u_{\eta},\mu)\leq\mathcal{L}_{\eta}(u_{\eta }, \lambda_{\eta}),\quad\forall\mu\in\Lambda, $$

which implies (8). Next, we will prove (7). Since

$$ \mathcal{L}_{\eta}(u_{\eta},\lambda_{\eta})\leq \mathcal{L}_{\eta }(v,\lambda_{\eta}),\quad\forall v\in X, $$

we use (37) to see that, for all $v\in X$,

$$\begin{aligned} \frac{1}{2}( u_{\eta},u_{\eta})_{X}- \frac{1}{2}( v,v)_{X}-(f_{\eta },u_{\eta}-v)_{X}+ \frac{m_{A}}{2L_{A}^{2}}B(u_{\eta}-v,\lambda_{\eta })+ \phi(u_{\eta})-\phi(v) \le0. \end{aligned}$$

For $\forall t\in(0,1)$, $v'\in X$ taking $v=u_{\eta}+t(v'-u_{\eta})$ in the above inequality, we obtain

$$\begin{gathered} -t\bigl( u_{\eta},v'-u_{\eta} \bigr)_{X}-\frac{t^{2}}{2}\bigl(v'-u_{\eta },v'-u_{\eta} \bigr)_{X}+ t\bigl(f_{\eta},v'-u_{\eta} \bigr)_{X} \\ \quad{}-\frac{m_{A}}{2L_{A}^{2}}tB\bigl(v'-u_{\eta}, \lambda_{\eta}\bigr)+\phi (u_{\eta})-\phi\bigl(u_{\eta}+ t \bigl(v'-u_{\eta}\bigr)\bigr) \leq0.\end{gathered} $$

Then, since *φ* is convex, we obtain

$$\begin{gathered} -t\bigl( u_{\eta},v'-u_{\eta} \bigr)_{X}-\frac{t^{2}}{2}\bigl(v'-u_{\eta },v'-u_{\eta} \bigr)_{X}+ t\bigl(f_{\eta},v'-u_{\eta} \bigr)_{X} \\ \quad{}-\frac{m_{A}}{2L_{A}^{2}}tB\bigl(v'-u_{\eta}, \lambda_{\eta}\bigr)-t\bigl(\phi \bigl(v'\bigr)- \phi(u_{\eta})\bigr)\leq0.\end{gathered} $$

Dividing by *t* and passing to the limit as $t\rightarrow0$, we get (7). This completes the proof of the lemma. □

### Lemma 23

*Problem* 9 *has a unique solution* $(u_{\eta},\lambda _{\eta})\in X\times\Lambda$.

### Proof

By (5), it is easy to verify that the map $v\mapsto\mathcal {L}_{\eta}(v,\mu)$ is convex and l.s.c. for all $\mu\in\Lambda$, and $\mu\mapsto\mathcal{L}_{\eta}(v,\mu)$ is concave and u.s.c. for all $v\in X$. Since *ϕ* is convex and l.s.c., it admits an affine minorant, see e.g. [18, Proposition 5.2.25], that is, there are $l\in X^{*}$ and $b\in\mathbb{R}$ such that

$$ \phi(v)\ge \langle l, v\rangle_{X} + b \quad\mbox{for all } v\in X. $$

Then we have

$$\begin{aligned} \mathcal{L}_{\eta}(v,0_{Y}) &=\frac{1}{2}(v,v)_{X}-( f_{\eta},v)_{X}+ \frac {m_{A}}{2L_{A}^{2}}B(v-w,0)+\phi(v) \\ &\ge\frac{1}{2}\|v\|_{X}^{2}-( f_{\eta},v)_{X}+ \frac {m_{A}}{2L_{A}^{2}}B(v-w,0)+\langle l, v\rangle_{X} + b.\end{aligned} $$

Therefore,

$$ \lim_{\|v\|_{X}\rightarrow\infty,v\in X}\mathcal{L}_{\eta }(v,0_{Y})= \infty. $$

Next, we will prove that

38 $$ \lim_{\|\mu\|_{Y}\rightarrow\infty,\mu\in\Lambda}\inf_{v\in X} \mathcal{L}_{\eta}(v,\mu)=-\infty. $$

From Lemma 4.2 and Corollary 4.6 in [19], for every $\mu\in \Lambda$ the following inequality:

39 $$ ( u_{\mu},v-u_{\mu})_{X}+ \frac{m_{A}}{2L_{A}^{2}}B(v-u_{\mu},\mu)+\phi (v)-\phi(u_{\mu})\ge( f_{\eta},v-u_{\mu})_{X}, \quad\forall v\in X $$

has a unique solution $u_{\mu}\in X$. It is easy to verify that

$$ \inf_{u\in X}\mathcal{L}_{\eta}(u,\mu)= \frac{1}{2}( u_{\mu },u_{\mu})_{X}-(f_{\eta},u_{\mu})+ \frac{m_{A}}{2L_{A}^{2}}B(u_{\mu }-w,\mu)+\phi(u_{\mu}). $$

Taking $v=0_{X}$ in (39), we obtain

$$ \|u_{\mu}\|_{X}^{2}+\frac{m_{A}}{2L_{A}^{2}}B(u_{\mu}, \mu)+\phi (u_{\mu})-( f_{\eta},u_{\mu})_{X} \le\phi(0). $$

Hence,

40 $$ \inf_{u\in X}\mathcal{L}_{\eta}(u,\mu)\leq \varphi(0)-\frac {1}{2}\|u_{\mu}\|_{X}^{2}+ \frac{m_{A}}{2L_{A}^{2}}L_{B}\|w\|_{X}\|\mu\|_{Y}. $$

By Lemma 8 we deduce that inequality (39) is equivalent to the following variational equation:

41 $$ ( u_{\mu},v)_{X}+\frac{m_{A}}{2L_{A}^{2}}B(v,\mu)+ \bigl(\nabla\phi(u_{\mu }),v\bigr)= ( f_{\eta},v)_{X}, \quad\forall v\in X. $$

From (4)(b) we deduce that

$$ \alpha_{B}\|\mu\|_{Y}\leq\sup_{v\in X,v\neq0_{X}} \frac{B(v,\mu )}{\|v\|_{X}}, $$

and combining with (41) we obtain

$$\begin{aligned} \frac{m_{A}\alpha_{B}}{2L_{A}^{2}}\|\mu\|_{Y}&\leq\sup_{v\in X,v\neq0_{X}} \frac{ (f_{\eta},v)_{X}-( u_{\mu},v)_{X}-(\nabla\phi (u_{\mu}),v)}{\|v\|_{X}} \\ &\leq \|f_{\eta}\|_{X}+\|u_{\mu}\|_{X}+ \|\nabla\phi(u_{\mu})\|_{X}.\end{aligned} $$

It follows from (5) that

$$ \big\| \nabla\phi(u_{\mu})\big\| _{X}\le L_{\phi} \|u_{\mu}\|_{X}+\big\| \nabla\phi (0)\big\| _{X}. $$

Therefore, there exists $c>0$ such that

42 $$ \|\mu\|_{Y}^{2}\leq c\bigl(\|f_{\eta} \|_{X}^{2}+\|u_{\mu}\|_{X}^{2} \bigr). $$

Then, combining (40) and (42), we deduce that there exists $c'>0$ such that

$$ \inf_{u\in X}\mathcal{L}_{\eta}(u,\mu) \leq-c'\bigl(\|\mu\|_{Y}^{2}-\| f_{\eta} \|_{X}^{2}\bigr)+\frac{m_{A}}{2L_{A}^{2}}L_{\varphi}\|w \|_{X}\|\mu \|_{Y}. $$

Since $\mu\in\Lambda$ is arbitrary, passing to the limit as $\|\mu\| _{Y}\rightarrow\infty$ we get (38). By applying Theorem 4 we deduce that the functional $\mathcal{L}$ has at least one saddle point, and then we conclude Problem 9 has at least one solution by applying Lemma 22.

Finally, we will show the uniqueness of the solution. In fact, let $(u_{\eta}^{1},\lambda_{\eta}^{1}),(u_{\eta }^{2},\lambda_{\eta}^{2})\in X\times\Lambda$ be two solutions of (41). Then we have

$$\begin{aligned} \bigl( u_{\eta}^{1}-u_{\eta}^{2},v \bigr)_{X}+\frac{m_{A}}{2L_{A}^{2}}B\bigl(v,\lambda _{\eta}^{1}- \lambda_{\eta}^{2}\bigr)+\bigl(\nabla\phi\bigl(u_{\eta}^{1} \bigr)-\nabla \phi\bigl(u_{\eta}^{2}\bigr),v \bigr)_{X} =0,\quad\forall v\in X. \end{aligned}$$

Choosing $v=u_{\eta}^{2}-u_{\eta}^{1}$ in the above equation, we get

43 $$ \bigl( u_{\eta}^{1}-u_{\eta}^{2},u_{\eta}^{2}-u_{\eta}^{1} \bigr)_{X} +\frac{m_{A}}{2L_{A}^{2}}B\bigl(u_{\eta}^{1}-u_{\eta}^{2}, \lambda_{\eta }^{2}-\lambda_{\eta}^{1}\bigr) + \bigl(\nabla\phi\bigl(u_{\eta}^{1}\bigr)-\nabla\phi \bigl(u_{\eta}^{2}\bigr),u_{\eta }^{2}-u_{\eta}^{1} \bigr)_{X}= 0. $$

From (8) it follows that

44 $$ B\bigl(u_{\eta}^{1}-u_{\eta}^{2}, \lambda_{\eta}^{2}-\lambda_{\eta }^{1}\bigr) \leq0. $$

Combining (5)(c), (43) and (44), we conclude that $u_{\eta}^{1}=u_{\eta}^{2}$. Moreover, we have

$$ B\bigl(v,\lambda_{\eta}^{1}-\lambda_{\eta}^{2} \bigr)=0, \quad\forall v\in X. $$

By (4)(b), we conclude that $\lambda_{\eta}^{1}=\lambda _{\eta}^{2}$. □
